# The Marginal Division of the Striatum and Hippocampus Has Different Role and Mechanism in Learning and Memory

**DOI:** 10.1007/s12035-014-8891-6

**Published:** 2014-10-02

**Authors:** Si-Yun Shu, Gang Jiang, Qi-Yi Zeng, Bin Wang, Hong Li, Lin Ma, Harry Steinbusch, Cai Song, Wood-Yee Chan, Xu-Hong Chen, Yon-Ming Wu, Rong Bao, Yan-chen Chen, Jang-Yen Wu

**Affiliations:** 1 Center of Pediatrics, Zhujiang Hospital, Southern Medical University, Guangzhou, Gunagdong 510282 China; 2 Department of Ear, Nose and Throat, Zhujiang Hospital, Southern Medical University, Guangzhou, Guangdong 510282 China; 3 Department of Radiology, PLA General Hospital, 28 Fuxing Road, Beijing, 100853 China; 4 Department of Translational Neuroscience, Faculty of Health, Medicine and Life Sciences, Maastricht University, P.O. Box 616, 6200 MD Maastricht, The Netherlands; 5 Research Institute for Marine drugs and Nutrition, College of Food Science and Technology, Guangdong Ocean University, Guangdong Province, China; 6 School of Biomedical Sciences, Faculty of Medicine, The Chinese University of Hong Kong, Hong Kong, China; 7 Central Laboratory of Shenzhen Second People’s Hospital, Shenzhen, Guangdong 518000 China; 8 Department of Neurology, Nanfang Hospital, Southern Medical University, Guangzhou, Guangdong 510515 China; 9 Department of Pediatrics, Sun Yet-San Hospital, Zhong Shan University, Guangzhou, 510120 China; 10 Florida Atlantic University, Charles E. Schmidt College of Medicine, 777 Glades Road, P.O. Box 3091, Boca Raton, FL 33431-0991 USA

**Keywords:** Hippocampal formation, The ventromedial area of striatum, The marginal division of neostriatum, MrD, pCREB, NMDA receptors, Memory function

## Abstract

The memory function of the hippocampal formation (Hip) and the marginal division (MrD) of neostriatum was compared. Rats with bilateral lesions of the MrD either immediate or 24 h after training in Y-maze were found to have decrease in correct runs in both groups. However, animals with transected afferent and efferent nerve bundles to isolate the Hip immediately or 24 h after training in Y-maze were found to show a decrease in correct runs only in the group injured immediately after Y-maze training but not in the 24 h group suggesting that MrD is likely involved in the entire process of long-term memory consolidation whereas the Hip only contributes to memory in the early stage. In addition, animals treated with a NMDA receptor (NMDAR) blocker, e.g. MK-801, showed decreased correct runs in Y-maze test and in expression level of phosphorylated CREB (pCREB) in neurons of the MrD but not in the Hip. Furthermore, animals treated with okadaic acid (OA), a potent protein phosphatase 1 inhibitor, showed increased correct runs in the Y-maze test. The expression level of pCREB and c-Fos and c-Jun was found increased in neurons of the MrD and the Hip in response to OA treatment. In conclusion, NMDAR and pCREB are involved in memory functions of both the Hip and the MrD. NMDAR might regulate pCREB level in neurons of the MrD but not in the Hip. Hence, the processes and mechanism of learning and memory involved in the MrD and the Hip may be different.

## Introduction

Since Scoville and Milner [1] reported the persistent impairment of short-term memory following bilateral medial temporal lobe resection in humans, the hippocampus (Hip) has been well accepted to play an important role in learning and memory. Since then, more brain regions have been found to be involved in learning and memory, such as the prefrontal cortex, amygdala, limbic system, and basal nucleus of Meynert (BNM) [2–4]. Previously, we have identified a new subdivision of the neostriatum, namely marginal division (MrD), which is situated between the ventromedial border of the neostriatum and the dorsolateral edge of the globus pallidus and is connected to several memory-related structures in the brain including the amygdala, NBM, and prefrontal cortex [5]. Since our report of MrD, the existence and the specific function of the MrD have been supported by numerous immunohistochemical and physiological studies throughout the world. Among various functions, it is interesting that the MrD has been shown to be involved in associative learning and declarative memory in the rat and human brains [6, 7]. Knocking down NK1R activity in the MrD by using an antisense oligonucleotide against NK1R messenger RNA (mRNA) inhibited learning and memory of rats in a Y-maze behavioral test. Thus, NK1R is likely to mediate the role of the MrD in learning and memory [8].

It has been reported that neurotransmitter receptors on the cell membrane [e.g. *N*-methyl-D-aspartate receptors (NMDARs)], calcium/calmodulin-dependent kinase (CaMK), cAMP-dependent protein kinase (PKA), mitogen-activated protein kinase (MAPK), cAMP responsive element-binding protein (CREB) and immediate early genes (IEGs) all played important roles in the molecular events of short-term and long-term memory [9]. Among them, the transcription factor CREB is a key component of intracellular signaling that regulates a wide range of biological functions including learning and memory [10]. The phosphorylation of Ser 133 is a critical step in CREB activation, while putative CREB target genes are numerous, possibly over 100, and these include IEGs such as c-Fos and c-Jun and other genes that regulate neurotransmission, cell architecture, signal transduction, transcription, metabolism, etc. [10]. Other molecules involved in the processes of long-term potentiation (LTP) and learning and memory include different subtypes of the glutamatergic NMDARs [11]. Here, we present several lines of evidence to support the conclusion that NMDAR and phosphorylated CREB (pCREB) are involved in memory functions of both the Hip and the MrD and NMDAR might regulate pCREB level in neurons of the MrD but not in the Hip suggesting that the processes and mechanism of the memory involved in the MrD and the Hip may be different.

## Materials and Methods

### Materials

#### Animals

Male Sprague–Dawley rats (Beijing Research Center for Experimental Animals, Beijing, China) weighing 220–300 g were used in all experiments. They were housed at a constant temperature of 25 ° C with a 12 h light/12 h dark cycle and provided with food and water ad labium. All behavioral tests were conducted at approximately the same time each day and were performed in accordance with the Ethical Committee for Animal Experiments of South Medical University in China. An effort was made to minimize the number of sacrificed animals.

#### Chemicals and Fine Reagents

Primary antibodies were obtained from the following sources as indicated: c-Fos and c-Jun were purchased from Santa Cruz Biotechnology; Santa Cruz, CA, USA; pCREB was purchased from Cell Signaling Technology, Beverly, MA, USA. The avidin-biotin-peroxidase complex (ABC) kit was purchased from Vector Laboratories, Burlingame, CA, USA.

## Methods

### Behavioral Tests

#### Morris Water Maze Test

The Morris water maze test was performed as described [12] with some modifications. A circular water tank (150 cm in diameter and 45 cm height) was filled to a height of 30 cm with water at approximately 22 °C. Four points equidistant around the tank circumference were designated north, east, south, and west, and the tank was divided into four equal quadrants. A transparent platform (10 cm in diameter and 28.5 cm height) was placed 30 cm from the wall in the center of the quadrant designed northeast. Each rat started from one of the four starting positions, and the sequence of the positions was selected clockwise. In each training trial, the time taken to escape onto the hidden platform was recorded (escape latency), and the rat was permitted to stay on the platform for 60 s and after each training trial had a rest for 120 s. The average escape latency was calculated from four training trials. Each rat was trained two blocks of which each block contained four training trials a day for five successive days starting at 8 days after the injection or surgery. On the final training day, a probe test was carried out to examine whether the rats had learned the position of the platform. The number of times each rat swam through the place where the platform had been located during training in 2 min was measured (crossing number).

#### Statistical Analyses

SPSS program (v. 10.0, SSPS Inc, Chicago, Ill, USA) was used to analyze all data. Both intergroup one-way ANOVA and the least-significant difference (LSD) test were used to compare multiple groups and to compare averages between each pair of the groups, respectively.

#### The Light-Foot-Shock Avoidance Y-Maze Test

The light-foot-shock avoidance Y-maze test is a good method to test learning and memory function, especially on the associative learning and declarative memory of rats [13, 14]. The light-foot-shock avoidance reaction is based on the memory-related conditional reflex, so it could be used to test the associative learning and declarative memory of the animals. The Y-maze had three arms with metal wires on their bottom to deliver electric shocks and lights at their ends. When the foot-shock avoidance test began, one arm with the light on (light zone) is the shock-free area, whereas the other two arms with the light off (dark zone) are areas with electric shocks. Electric shocks can be delivered to any of these three arms during the test. Rats by nature all prefer to enter the dark zones at the beginning of the test. After receiving a foot-shock, most of the rats soon learnt to escape from the dark to the light zone to avoid electric shocks. After several shocks, rats learned and remembered that the light zone is a safe area, and hence, they ran directly to the light zone whenever the light is shifted from one area to the other. During the test, the rats are considered to be able to learn and remember the correct route of escape from the electric shock if they run to the light zone within 10 s after the light shifts from one arm to another arm of the Y-maze. The number of correct escapes (running to the light zone within 10 s) in 30 electric shocks is used to quantify memory functions of rats. All rats have been tested twice before carrying out the behavioral tests, and only those who have passed the first test with at least 10 correct escapes and have passed the second test with at least 15 correct escapes would be used in the behavioral tests. Rats in the training group were trained in the foot-shock avoidance Y-maze with simultaneous light stimulus changes for 30 electric shocks per day for three consecutive days, whereas rats in the pseudo-training group did not receive the simultaneous light stimulus changes when they were subjected to electric shock. The control group animals were held in the Y-maze without any stimuli for 20 min per day for 3 days.

#### Pharmacological and Surgical Interventions

All rats were anesthetized with 10 % chloral hydrate (3.5 ml/kg, i.p.). Animal heads were fixed on a stereotaxic apparatus, and the bregma was marked as the reference point according to the description in “The Rat Brain in Stereotaxic Coordinates” [15].

**Fig. 1:**
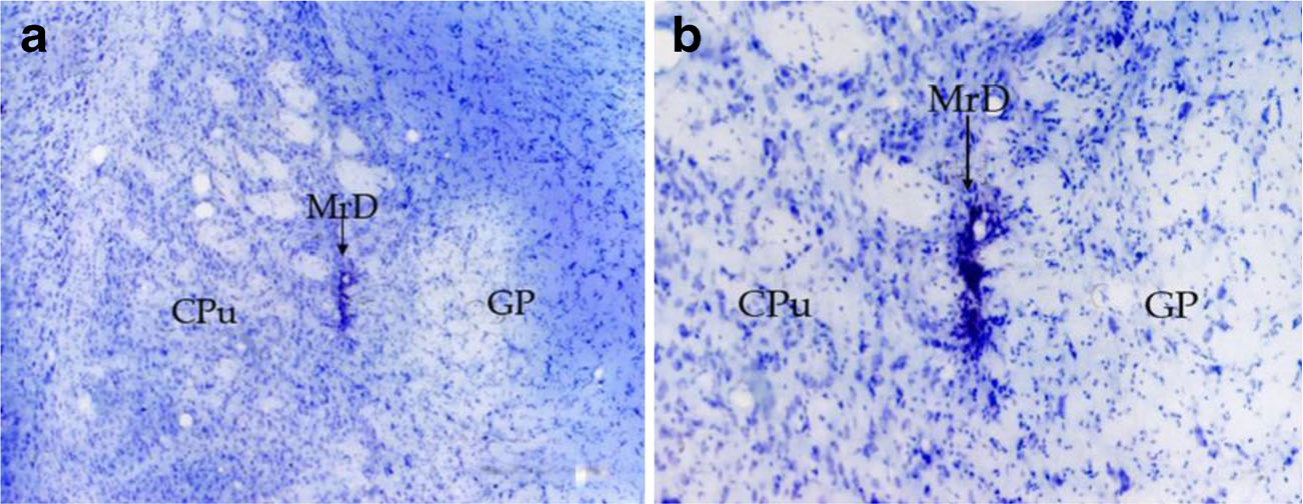
A representative picture showing the injection site of the MrD demonstrated by Nissl staining. **a** The injection site demonstrated by Nissl staining showing the accurate location of the MrD (*arrow*, ×40; *n* = 9 for kainic acid-lesioned group and *n* = 8 for the saline control group). **b** The higher magnification of A (*arrow*, ×100)

Kainic acid (0.2 μl, 0.1 %) was bilaterally microinjected into the MrDs of rats trained for 3 days in the Y-maze. The coordinates for the MrD injection were 1.6 mm posterior to the bregma, 4.2 mm lateral to the midline, and 5.5 mm ventral from the skull surface. Physiological saline (0.2 μl) was injected instead of kainic acid in the MrD for the control group rats. Microinjections were performed on rats either immediately or 24 h following the 3-day training regime in the Y-maze so that the early phase of memory consolidation (lesion group *n* = 9, saline control *n* = 8) or the more advance stage of consolidation and retrieval of memory process (lesion group *n* = 11, saline control *n* = 9) could be examined. The injected tract was examined by Nissl staining (Fig. 1) to determine whether the drug was accurately microinjected into the MrD. Only rats with successfull injections into the MrD were used for behavioral tests 6 days post-injection immediately (lesion group *n* = 10) or 24 h (*n* = 10) after a 3 day-Y-maze training the fornix/fimbria fiber bundle (FF) was bilaterally transected with a thin blade to create the Hip lesions. The coordinates for the FF transecting were 1.8 mm posterior to the bregma, 1 mm lateral from the midline, 4.5 mm from the brain surface after the skull was opened, and then 2 mm further outward [16]. The Hip lesioned group would also be used to examine early phase or more advanced stage of the memory consolidation process and retrieval. Sham-operated animals (*n* = 10) received the identical surgical treatment except that the cut was made more superficially at 2 mm deep instead of 4.5 mm from the brain surface to avoid injuring the FF. Behavioral tests were also performed 6 days after the surgery. For the Morris water maze test, rats were treated with kainic acid or subjected to bilateral FF transecting as described above for the Y-maze test except rats that were tested for the Morris water maze test 8 days after the treatment.

Okadaic acid (OA) (0.5 μl, 40 ng/μl) or equivalent volume of saline was microinjected bilaterally into the MrD and the lateral ventricles of the rats trained in Y-maze for 3 days. The coordinates for the lateral ventricles injection were 1.6 mm posterior to the bregma, 3.0 mm lateral to the mid line, and 3.3 mm ventral from the skull surface. Twelve hours after the OA injection, the rats were anesthetized and the brains were removed for immunohistochemistry as described below.

Similarly, MK-801 (0.2 μl, 1 %) or saline (0.2 μl) was also microinjected bilaterally using coordinates described above into the MrD of rats trained in the Y-maze for 3 days. Behavioral tests were also performed on the MK-801 (*n* = 10) or saline-injected (*n* = 8) rats, 6 days after the microinjection. In addition, MK-801 (0.01 %, 0.3 mg/kg, *n* = 5) was intraperitoneally injected to the rats trained in Y-maze for 3 days to examine the relationship between NMDAR and pCREB in the MrD and the Hip. Physiological saline was intraperitoneally injected instead of MK 801 in control group rats. Thirty minutes after the intraperitoneal injection of MK-801, the rats were anesthetized and the brains were removed for immunohistochemistry as described below.

#### Immunohistochemistry

Immunohistochemical identification of brain protein markers was conducted as described previously [17]. Briefly, rats were first anesthetized with chloral hydrate (10 %, 3.5 ml/kg) by intraperitoneal injection, followed by transcardial perfusion with ice-cold 4 % paraformaldehyde (500 ml) in 0.1 M phosphate buffer, pH 7.4, after a brief rinse with saline (100 ml). Brains were then removed after perfusion and were immersed in 0.1 M phosphate buffer, pH 7.4, with 30 % sucrose solution at 4 °C overnight. Brains were then sectioned coronally at 40 μm in a cryostat (Leica 1800, Germany), followed by incubation with primary antibodies to either c-Fos (1:600), c-Jun (1:600), or pCREB (1:200) for 40 h at 4 °C, and the sections were further incubated with the ABC and the glucose oxidase and nickel ammonium sulfate-intensified diaminobenzidine complex [18], which could strongly enhance the immunostaining. Negative controls were performed by omitting the primary antibodies.

#### In Situ Hybridization

Rats were deeply anesthetized with 10 % chloral hydrate (3.5 ml/kg, i.p.), followed transcardial perfusion with ice-cold 4 % paraformaldehyde in sodium borate buffer (pH 7.4, 4 °C) after a brief physiological saline rinse. Brains were removed and were immersed in 0.1 M phosphate buffer, pH 7.4, with 30 % sucrose solution at 4 °C overnight before sectioning coronally at 40 μm with a cryostat (Leica 1800, Germany). In situ hybridization, probes were designed according to Landwehrmeye et al. [19] and synthesized by TaKaRacompany (Japan). Sequences of the probes were as follows: NR1 5′-AAA CCA GAC GCT GGA CTG GGA GTA GGG CGG CAC CGT GCG AAG-3′; NR2A5′-AGA AGG CCC GTG GGA GCT TTC CCT TTG GCT AAG TTT C-3′; NR2B 5′-CAT GTT CTT GGC CGT GCG GAG CAA GCG TAG GAT GTT GGA GTG GGT-3′.

All probes were labeled with digoxigenin for hybridization detection according to the instruction of the manufacturer (Roche). After rinse, sections were first incubated in the pre-hybridization solution at 39 °C for 1.5 h followed by incubation in hybridization solution containing 1 μg/μl probe at 41 °C for 16 h. After brief rinse with buffer solution, the sections were further incubated in the anti-digoxingenin antibody solution for 1.5 h before treated with the chromogen. Positive hybridization products were expressed as a bright blue color.

#### Statistical Analyses

For immunohistochemistry studies, eight sections which covered the brain area of interest, e.g. hip or MrD from each rat, were quantified for statistical analyses in each group. The c-Fos, c-Jun, or pCREB immunopositive nuclei of neurons were measured separately. The average numbers of c-Fos, c-Jun, or pCREB immunopositive neurons were counted respectively at 20× under an Olympus microscope (AH3, Center Valley, PA, USA) and compared between each group. Results were expressed as means ± SEM. SPSS program (v. 13.0, SSPS Inc, Chicago, Ill, USA) was used to analyze all data. The intergroup one-way ANOVA was performed to compare multiple groups, and the independent *t* test was used to compare averages between two groups. In behavioral experiments, the average correct escapes from each group in the Y-maze tests were compared and results were expressed as means ± SEM. SPSS program (v. 13.0, SSPS Inc, Chicago, Ill, USA) was used to analyze all data. Both intergroup one-way ANOVA and the LSD test were used to compare multiple groups and to compare averages between each pair of the groups, respectively.

## Results

### Behavioral Tests

#### The Morris Water Maze Test

**Fig. 2:**
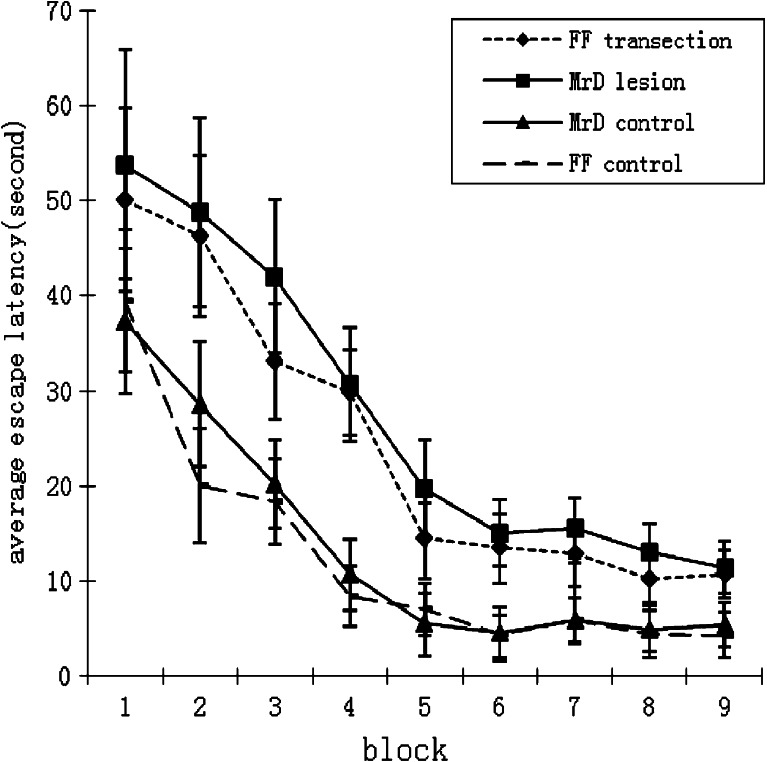
Effect of lesion of the marginal division (*MrD*) and transection of the fornix/fimbria fiber bundle (*FF*) on escape latency in water maze test. Four groups of rats including the MrD lesion group, FF-transected group and their respective control group were tested in Morris water maze test. The results are expressed as means ± SEM and plotted as the escape latency in seconds as a function of the number of the training trial in block (each block contains four training trials). For the MrD-lesioned groups, the escape latency in seconds for blocks 1 to 9 is 53.83 ± 9.62, 48.82 ± 8.47, 42.05 ± 6.25, 30.71 ± 4.51, 19.79 ± 3.84, 15.12 ± 3.46, 15.64 ± 3.17, 13.10 ± 2.90, 11.46 ± 2.48. For the FF-transected groups, the escape latency in seconds for blocks 1 to 9 is 50.14 ± 11.82, 46.33 ± 10.24, 33.08 ± 8.48, 29.92 ± 5.61, 14.48 ± 5.03, 13.50 ± 3.53, 12.95 ± 3.16, 10.27 ± 3.05, 10.72 ± 2.35, respectively. For the FF-transected groups, the escape latency in seconds for blocks 1 to 9 is 53.83 ± 9.62, 48.82 ± 8.47, 42.05 ± 6.25, 30.71 ± 4.51, 19.79 ± 3.84, 15.12 ± 3.46, 15.64 ± 3.17, 13.10 ± 2.90, 11.46 ± 2.48. For the MrD control group, the escape latency in seconds for blocks 1 to 9 is 37.33 ± 7.62, 28.58 ± 6.54, 20.21 ± 4.70, 10.78 ± 3.55, 5.56 ± 3.24, 4.60 ± 2.62, 5.89 ± 2.40, 5.05 ± 2.38, 5.53 ± 2.32. For the FF control groups, the escape latency in seconds for blocks 1 to 9 is 39.52 ± 7.44, 20.10 ± 6.07, 18.43 ± 4.49, 8.38 ± 3.22, 7.14 ± 2.70, 4.49 ± 2.63, 5.90 ± 2.48, 4.35 ± 2.42, 4.28 ± 2.21. *n* = 8; *p* < 0.01 between the lesioned group and the control groups while *p* > 0.05 between the MrD-lesioned and FF-transected groups

**Fig. 3:**
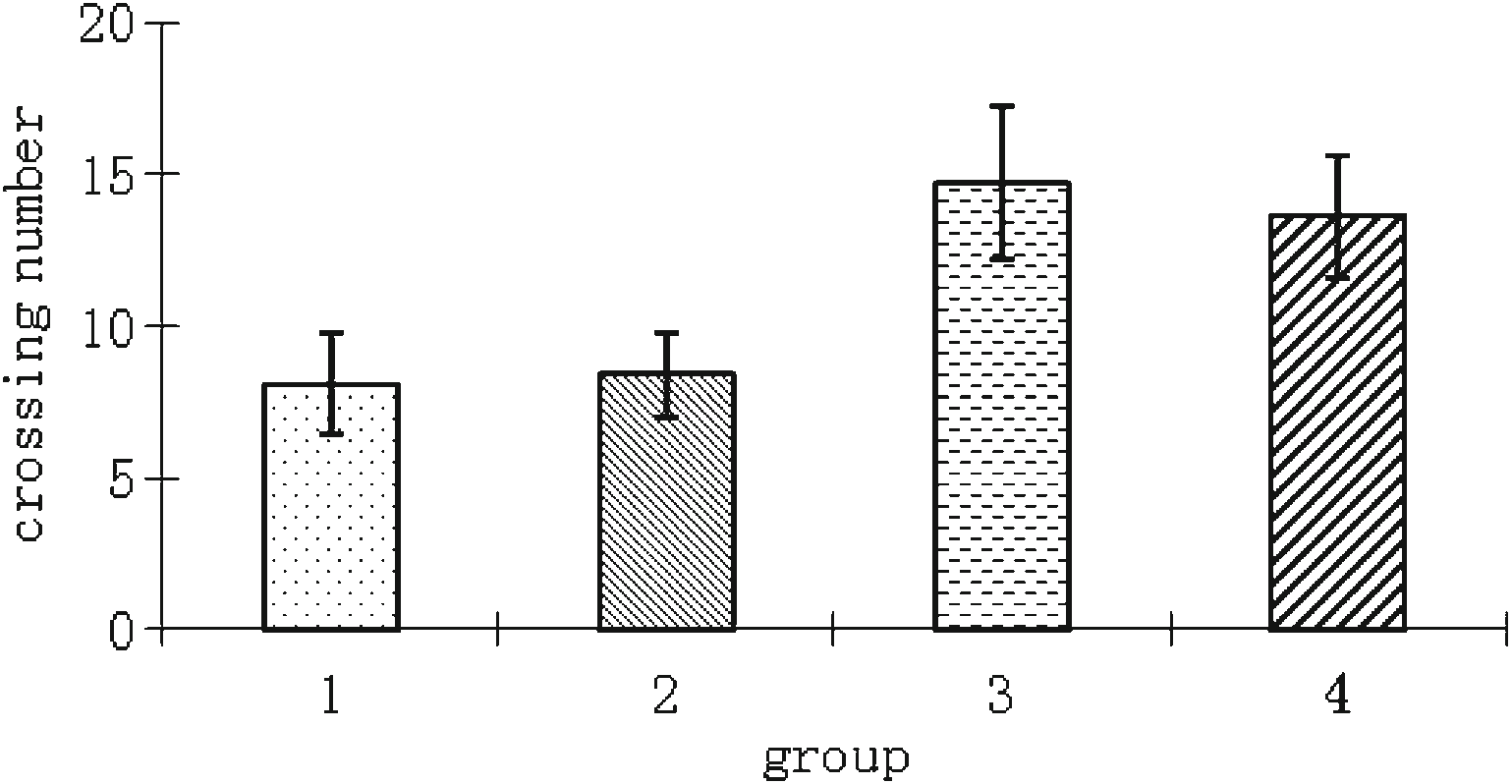
Effect of lesion of the marginal division (*MrD*) and transection of the fornix/fimbria fiber bundle (*FF*) on platform crossing in water maze test. The number of platform crossing among the MrD lesioned, FF transected, and their respective control groups are expressed as means ± SEM. Groups 1 to 4 refer to lesion in the MrD, transection of FF, MrD control, and FF control group, respectively. Group 1 (MrD lesion): 8.1 ± 1.72; group 2 (FF transection) 8.4 ± 1.42; group 3 (MrD control) 14.7 ± 2.49; group 4 (FF control) 13.6 ± 2.06. *n* = 8; *p* < 0.01 between the lesioned group and the control groups while *p* > 0.05 between the MrD-lesioned and FF-transected groups

In the Morris water maze test, the escape latency which is a measurement of learning acquisition for both the MrD-lesioned and the FF-transected rats increases significantly throughout the entire nine test blocks starting from 37.33 to 39.52 s, the escape latency for the control groups, to 53.83 and 50.14 s, (*p* < 0.01). respectively, in the first block of test to 11.46 and 10.72 s for the MrD-lesioned and FF-transected group in the block 9 test compared to 4.28 and 5.53 for the MrD and FF-transection control, respectively, suggesting impairment of learning acquisition in both the MrD-lesioned and FF-transected groups as shown in Fig. 2. However, the average escape latency between the two treated groups, MrD lesioned and FF transected, throughout the entire nine block tests is insignificant (*p* > 0.05) suggesting that the mechanism involved in learning acquisition for both the MrD and Hip is similar. In addition to the results of escape latency, the results of the crossing numbers during the spatial probe tasks, a measurement of memory retrieval, also show impairment of memory retrieval in the MrD-lesioned and FF-transected group as shown in Fig. 3, in which the numbers of crossing platform of two treated groups during the spatial probe tasks were both decreased from 14.7 and 13.6 to 8.1 and 8.4, respectively, the crossing number for the control groups, (*p* < 0.01). The decreasing levels for both the MrD-lesioned and FF-transected group were similar (*p* > 0.05) suggesting that the mechanism involved in the spatial memory retrieval of water maze in MrD and Hip is similar.

#### The Light-Foot-Shock Avoidance Y-Maze Test

When the lesion of MrD and transection of FF was done immediately after 3-day training period, the performance of the MrD-injured and FF-transected rats in the Y-maze test was found to be poorer than that before the lesions and poorer than that of the sham control animals. The number of correct escapes to the light zone was significantly decreased in the animals with lesions either in the MrD or FF, decreasing from 18.56 ± 2.30 before lesion to 10.11 ± 3.89 after lesion for the MrD group, vs 19.00 ± 2.05 before FF transection to 12.80 ± 3.99 after FF transection with *p* < 0.01. No significant difference was found in the number of correct escapes before or after the lesion or transection in both the MrD and FF groups (Table 1). These observations demonstrated that the MrD or FF were both involved in the early phase of the memory consolidation process. However, when the lesions were made 24 h after rats were trained in the Y-maze for 3 days, only the MrD-injured rats performed more poorly, decreasing from 18.27 ± 3.07 before lesion to 9.27 ± 4.29 after lesion with *p* < 0.01 while the performance of the FF-transected rats was found to be unaffected by FF transection since the number of correct escapes before and after transection is about the same, 19.90 ± 3.84 before and 21.10 ± 4.68 after the transection of FF. Again, the sham group of both the MrD and FF groups shows no significant difference in the number of correct escape (Table 2). These results suggested that the MrD may play a more important role than Hip in the more advanced stage of associative learning and declarative memory while the Hip may be not involved in the advanced phase of memory process (Table 3).

**Table 1:** Number of correct escapes to the light zone of the Y-maze before and after lesions of the MrD and FF†

Group	*N*	Number of correct escapes*	
		Before lesion	After lesion
MrD lesioned	9	18.56 ± 2.30	10.11 ± 3.89**
MrD sham	8	19.20 ± 3.02	22.63 ± 3.58
FF transected	10	19.00 ± 2.05	12.80 ± 3.99**
FF sham	10	18.70 ± 2.75	20.60 ± 4.88

†Lesion was made immediately after the 3-day training in the Y-maze

*Values are expressed as mean ± SE

**p < 0.01 when compared with the value for the corresponding sham control group

**Table 2:** Number of correct escapes to the light zone of the Y-maze before and after lesions of the MrD and FF†

Group	*N*	Number of correct escapes*	
		Before lesion	After lesion
MrD lesioned	11	18.27 ± 3.07	9.27 ± 4.29**
MrD sham	9	20.33 ± 4.39	22.56 ± 4.25
FF transected	10	19.90 ± 3.84	21.10 ± 4.68
FF sham	10	19.70 ± 4.64	22.00 ± 4.89

†Lesion was made 24 h after the 3-day training in the Y-maze

*Values are expressed as mean ± SE

**p < 0.01 when compared with the value for the corresponding sham control group

**Table 3:** Comparison of the MrD and the Hip on learning and memory after lesion in rats based on Y-maze test

	Time of Y-maze test	
Lesion location	Immediately after lesion	24 h after lesion
MrD	Depressed memory	Depressed memory
Hip	Depressed memory	Unaffected memory

#### Effects of Okadaic Acid and MK-801 on the Performance of Rats in Y-Maze Test

**Fig. 4:**
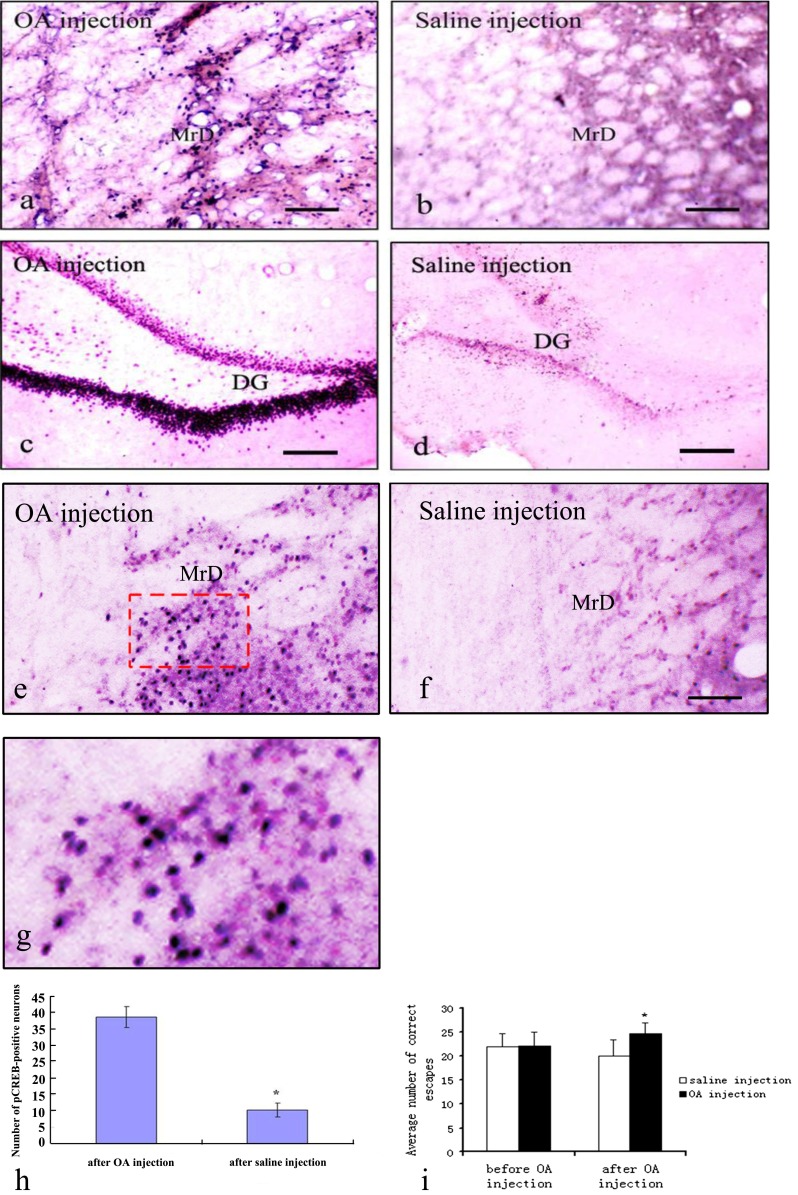
Effects of bilateral ventricular injection of okadaic acid (*OA*) on the expression of pCREB and Y-maze performance in rats. The expression of pCREB (**a**–**e**) was increased by bilateral ventricular injection of OA, and the performance in the Y-maze was improved after the bilateral injection of OA directly into the MrD (**f**). **a** A representative staining pattern with anti-pCREB antibody of brain section obtained from the rats that had received bilateral ventricular injection of OA, inhibitor of phosphatase. The pCREB-positive nuclei (*stained blue*) of the fusiform neurons (oriented dorsolaterally) were markedly increased in the MrD. **b** Same as in **a** except that the brain section was obtained from the rats that had received bilateral ventricular injection of saline as control. Very few p-CREB-positive neurons were observed. **c** Same as in **a** except the brain section used was in the Hip region instead of the MrD region. Similarly, the number of pCREB-positive granular neurons of the Hip in rat brain is also increased dramatically in the OA-injected group. **d** Same as in **c** except that the brain section was obtained from the rats that had received bilateral ventricular injection of saline as control. Very few p-CREB-positive neurons were observed. *Scale bar*: 100 μm. **e** Another section as in **a. f** Another section in **b. g** The same as in **e** except that the area marked with *red dash line* was magnified at 20× for cell counting. **h** Bar chart showing average number of pCREB-positive neurons in the MrD expressed as means ± SEM after trained in the Y-maze for the OA-injected group, 38.12 ± 3.02 and the saline-injected group 10.05 ± 1.98, respectively (*t = −19.74, *p* < 0.01 when the numbers of pCREB-positive neurons were compared with the saline-injected group by independent *t* test). **i** Bar chart showing the performance of the rats in the foot-shock avoidance Y-maze test expressed as means ± SE of the average number of correct escape before and after the bilateral injection of OA directly into the MrD. Before injection, 22.00 ± 3.10 (OA control group), 21.83 ± 2.92 (saline control); after injection, 24.71 ± 2.21 (OA-injected group), 20.00 ± 3,46 (saline-injected group). *n* = 6; *t = −3.92, *p* < 0.01 when compared with the saline-injected control group by independent *t* test

**Fig. 5:**
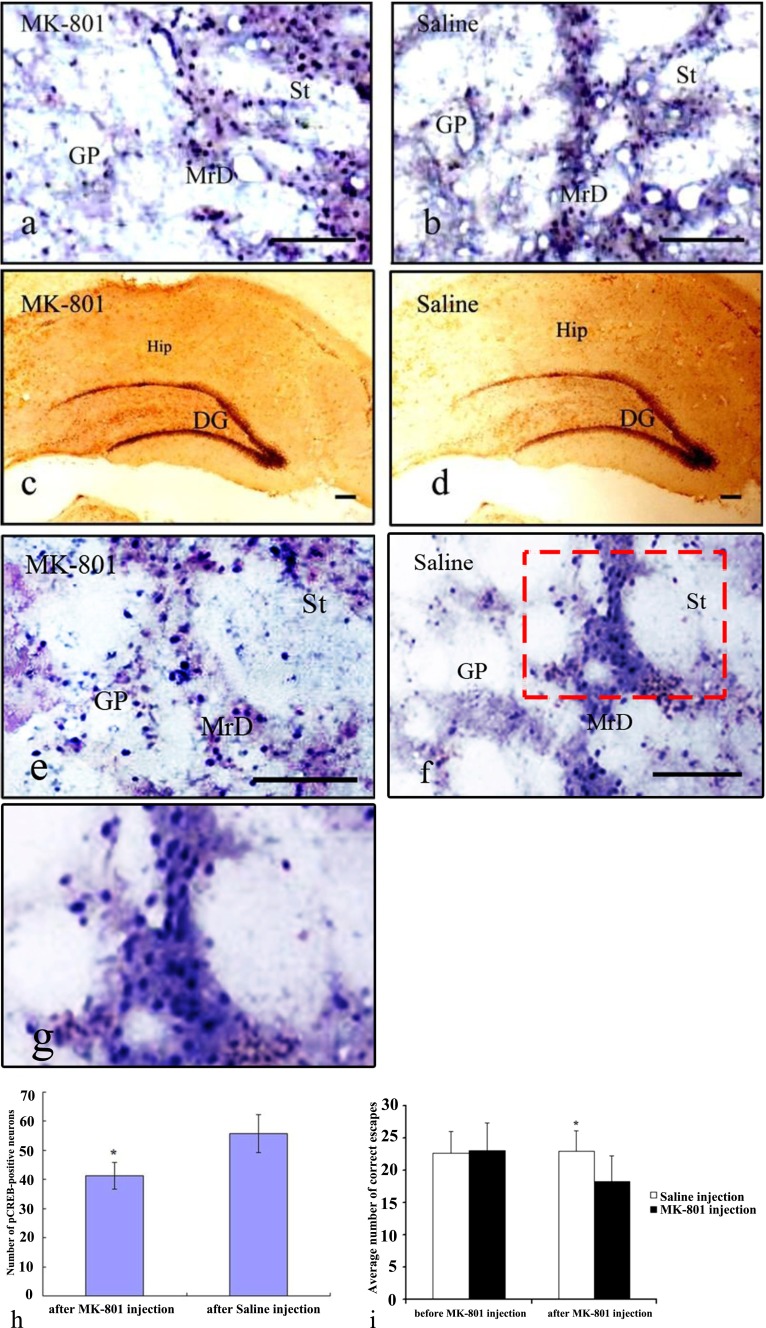
Effects of MK-801 on the expression of pCREB and Y-maze performance in rats. **a** A representative staining pattern with anti-pCREB antibody of brain section obtained from the rats that had received the intraperitoneal injection of MK-801, a blocker of NMDR receptor. **b** Same as in **a** except that the brain section was obtained from the rats that had received the intraperitoneal injection of saline as control. The pCREB-positive nuclei (*stained blue*) of the fusiform neurons (oriented dorsolaterally) was decreased in the MrD in the MK-801-injected group (**a**) as compared with the group injected with normal saline (**b**). **c** Same as in **a** except that the brain section used was in the Hip region instead of the MrD region. **d** Same as in **c** except that the brain section was obtained from the rats that had received the intraperitoneal injection of saline as control. The number of pCREB-positive granular neurons of the hippocampus formation was similar to the MK-801-injected group **c** as compared with the saline-injected group **d. e** Another section as in **a. f** Another section in **b**. *Scale bar*: 100 μm. **g** The same as in **f** except that the area marked with *red dash line* was magnified at 20× for cell counting. **h** Bar chart showing the average numbers of pCREB-positive neurons in the MrD expressed as means ± SEM after the foot-shock avoidance training in the Y-maze for the MK-801-injected group, 41.30 ± 4.67 and the saline-injected group, 55.80 ± 6.58 (*t = 4.92, *p* < 0.01 as compared to the control by independent *t* test). **i** Bar chart showing the performance of the rats in the Y-maze expressed as means ± SE of the average number of correct escape before and after the bilateral injection of MK-801 directly to the MrD. Before injection, 23.10 ± 4.23 and 22.63 ± 3.34 for MK-801and saline control group, respectively; after injection, 18.30 ± 3.92 and 23.00 ± 3.12 for MK-801- and saline-injected group, respectively. *n* = 8; *t = −3.08, *p* < 0.01 when compared with the saline-injected group by independent *t* test

When OA, a protein phosphatase inhibitor, was bilaterally microinjected into the MrD of rats (*n* = 8), the average number of correct escapes was significantly increased from 22 to 25 by comparison to the saline-injected group (*n* = 6) (*t* = −3.92 and *p* < 0.01) as shown in Fig. 4i. On the other hand, when MK-801( a NMDAR blocker) was microinjected to the MrD of rats (*n* = 10), the average number of correct escapes in the Y-maze was significantly decreased from 23 to 18 as compared with that of saline-injected animals (*n* = 8) (*t* = −3.08 and *p* < 0.01) (Fig. 5i). These results suggest that phosphorylation of CREB and glutamate neurotransmission, particularly the NMDA system, are likely to be involved in the associative learning and declarative memory process in rat as measured in the Y-maze test.

#### Effects of Okadaic Acid and MK-801on the Expression of pCREB in Marginal Division and Hippocampus

The number of pCREB-positive nuclei (stained blue) of the fusiform neurons (oriented dorsolaterally) was found to be markedly increased in the MrD of the group which had received bilateral ventricular injection of OA (Fig. 4a, e) as compared with the saline-injected animals (Fig. 4b, f). Similarly, the number of pCREB-positive granular neurons of the Hip in rat brain is also increased dramatically in the OA-injected group (Fig. 4c) as compared with the saline-injected group (Fig. 4d). Upon counting of pCREB-positive neuron using a higher magnification power as illustrated in Fig. 4g which is 20× magnification of those in Fig. 4e, f, we found that the treatment with OA markedly increases the number of pCREB-positive neurons by 3.8-fold, from 10 to 38 as shown in Fig. 4h. On the contrary, a decrease of pCREB expression in the nuclei (stained blue) of the fusiform neurons (oriented dorsoventrally) of the MrD was observed in the MK-801-treated group (Fig. 5a) as compared with the group injected with saline (Fig. 5b). However, the number of pCREB-positive granular neurons of the hippocampus formation in the MK-801-injected group (Fig. 5c) was not significantly different from that of the saline-injected group (Fig. 5d). Upon cell counting at higher magnification power as illustrated in Fig. 5g which is 20× magnifications of those in Fig. 5e, f, the number of pCREB-positive neurons in the MrD decreases about 25 % from 55 to 41 after treatment with MK-801 compared with the saline-injected group (Fig. 5h).

#### Effect of Training on the Expression of c-Fos, c-Jun, and pCREB in the Marginal Division and the Hippocampus

**Fig. 6:**
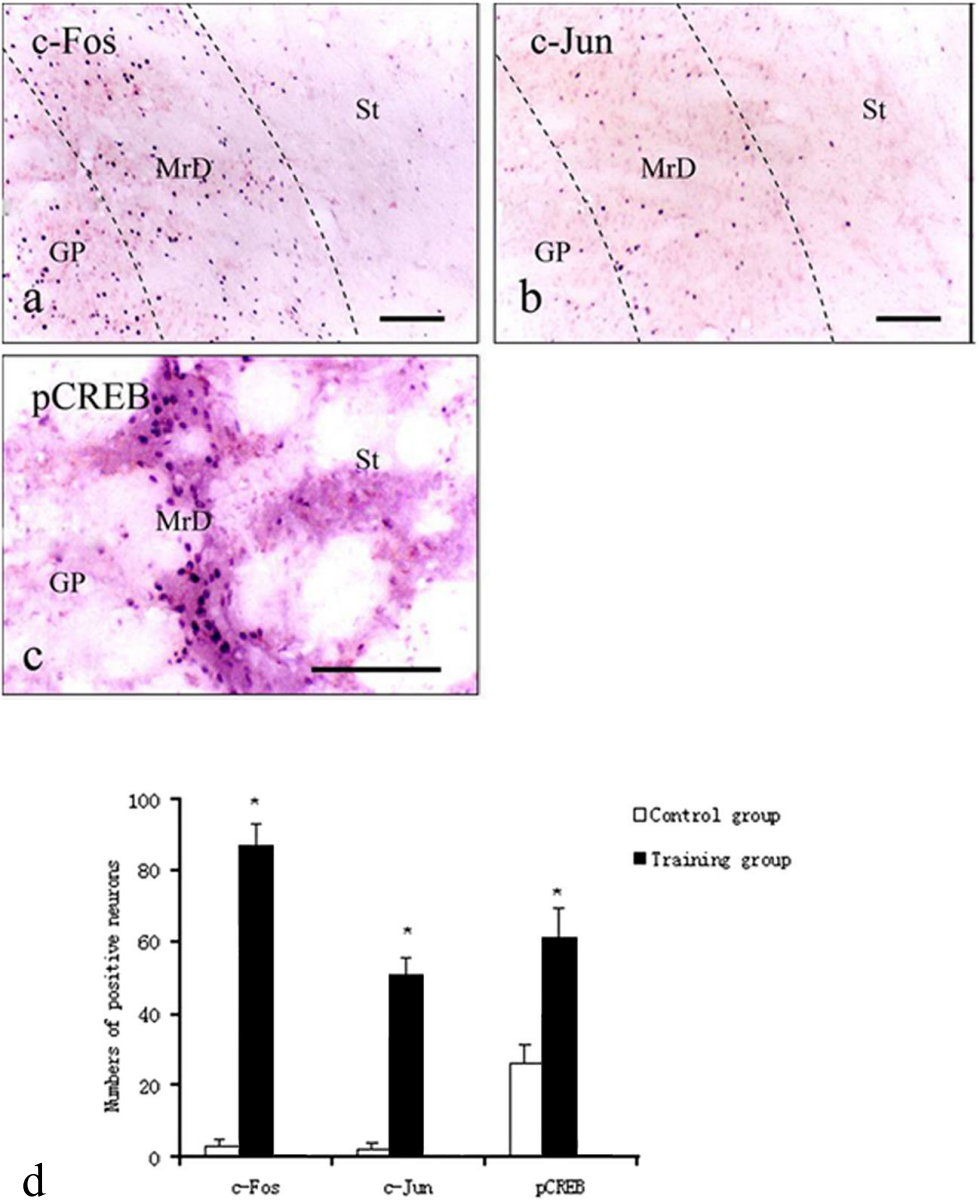
Immunohistochemical labeling of two immediate early genes (IEGs) c-Fos and c-Jun and pCREB in the MrD of the neostriatum after rats trained in the Y-maze for 3 days. The numbers of c-Fos (**a**), c-Jun (**b**) and pCREB (**c**) positive nuclei of the fusiform neurons of the MrD (stained with a *purple blue color* with their long axis oriented dorsoventrally) were increased as compared with the numbers before training. *Scale bar*: 100 μm (the length of bars showed the different magnification of each figure. The number of cells was counted by area of the section). *St* Striatum, *GP* globus pallidus, *DG* hippocampal dentate gyrus. **d** Average numbers of c-Fos, c-Jun, and pCREB-positive neurons in the MrD expressed as means ± SE after the foot-shock avoidance training in the Y-maze for the control and training groups. For pCREB, the number is 24.30 ± 5.66 and 61.80 ± 7.76 for the control and the training group, respectively; for c-fos and c-Jun, the number is 3.3 ± 1.89 and 87.50 ± 5.84 and 2.5 ± 1.58 and 50.80 ± 4.94, respectively, for the control and the training group. (*p* < 0.01 when the numbers of c-Fos, c-Jun, and pCREB-positive neurons were compared with the control values, *F* = 1,283.68, 733.36, and 194.18 respectively by the one-way ANOVA)

**Fig. 7:**
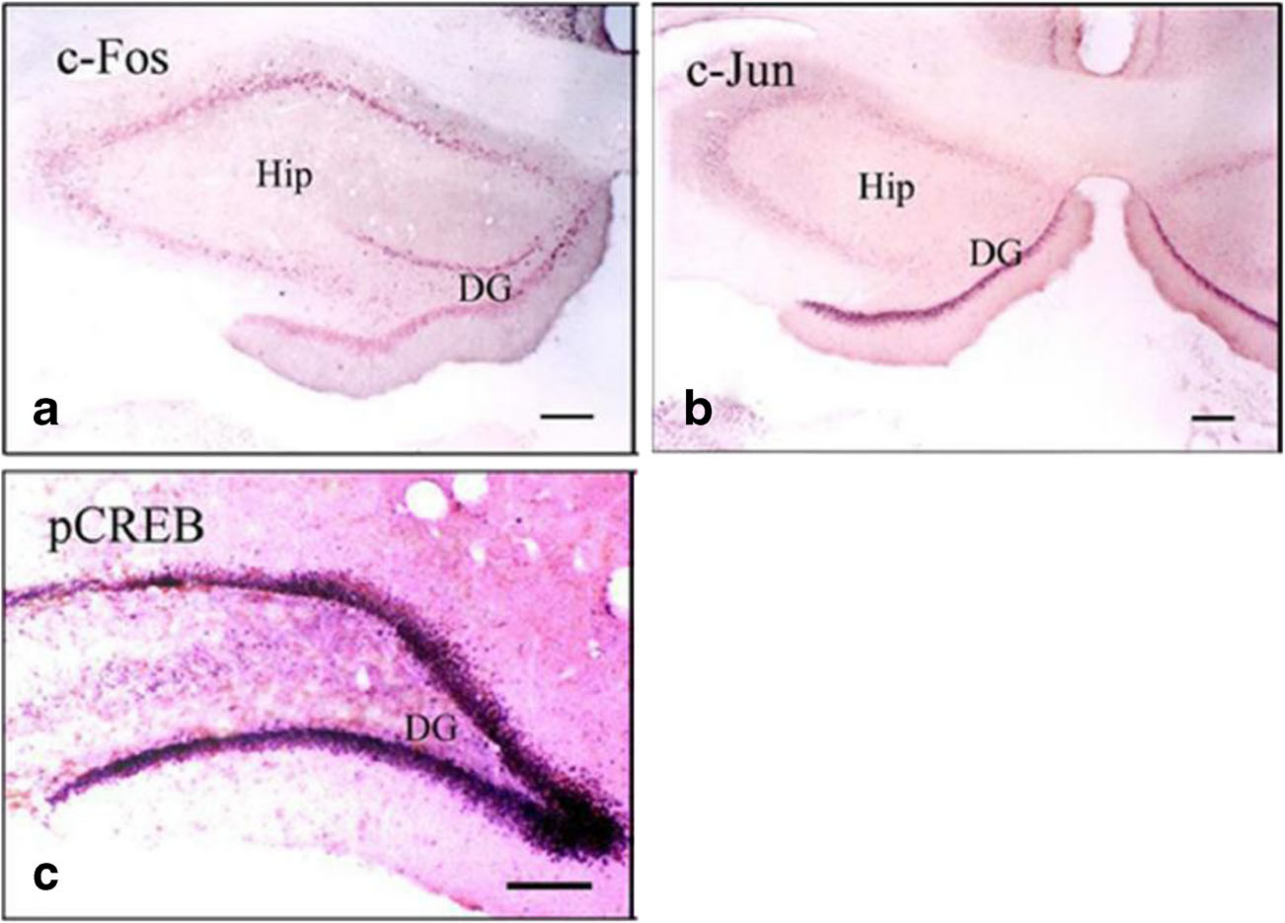
Immunohistochemical labeling of two immediate early genes (IEGs) c-Fos and c-Jun and pCREB in the Hippocampus (Hip) after rats trained in the Y-maze for 3 days. The numbers of c-Fos (**a**) and c-Jun (**b**) and pCREB (**c**) positive nuclei of the granular cells of the Hip (stained in purple blue) were increased as compared with the numbers before training. *Scale bar*: 100 μm (The length of bars showed the different magnification of each figure). *DG* hippocampal dentate gyrus

Results of immunohistochemical studies showed that when the rats were trained in Y-maze for 3 days, the protein level of the two immediate early genes (IEGs), c-Fos and c-Jun, as well as pCREB were markedly increased in both the MrD (Fig. 6a, b, c) and the Hip (Fig. 7a, b, c). Positive immunoreactive products appear as a purple blue color in the nuclei of neurons. There were very few neurons that showed positive immunostaining in the MrD and the Hip of the control group (data not shown). The number of neurons showing positive staining for c-Fos, c-Jun, and pCREB in the pseudo-training group is between the Y-maze trained and the control groups (data not shown). Upon cell counting, the number of c-Fos-, c-Jun-, and pCREB-positive neurons in the MrD in the Y-maze-trained group increases from 3.3, 2.5 and 24 to 87, 50 and 61, respectively, representing a 23-, 20-, and 2.5-fold more than that of the control group, respectively (*n* = 8; *p* < 0.01 when the numbers of c-Fos, c-Jun, and pCREB-positive neurons were compared with the control values, *F* = 1,283.68, 733.36, and 194.18, respectively, by the one-way ANOVA, Fig. 6d).

#### Localization of mRNA Expression of NMDAR1, NMDAR2A, and NMDAR2B in the Marginal Division and the Hippocampus by In Situ Hybridization

**Fig. 8:**
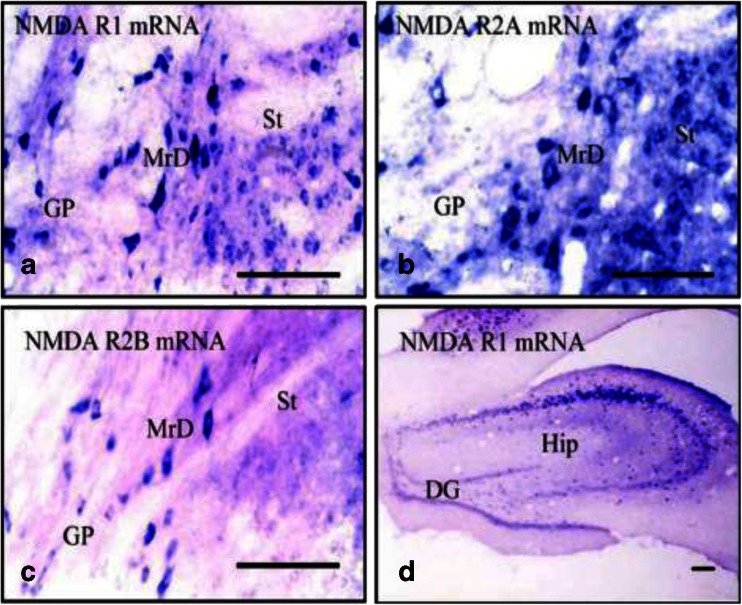
Localization of mRNA expression of NMDAR1, NMDAR2A, and NMDAR2B in the fusiform neurons by in situ hybridization. mRNA expression of NMDAR1 (**a**) NMDAR2A (**b**) and NMDAR2B (**c**) in the fusiform neurons (the positive-stained mRNA in a *blue color* and the fusiform neurons oriented dorsoventrally) of the MrD and mRNA expression of NMDAR1 (**d**) in the granular neurons of the hippocampus formation as demonstrated by in situ hybridization (the positive-stained mRNA shown in *blue color*). *St* striatum, *GP* globus pallidus, *DG* hippocampal dentate gyrus. *Scale bar*: 100 μm

Expression of mRNA of the NMDAR subunits, NMDAR1, NMDAR2A, and NMDAR2B, was detected in the MrD by in situ hybridization using the DNA probe with sequence specific to each NMDAR subunit as described in “Materials and Methods”. A representative picture of in situ hybridization is shown in Fig. 8. Results from in situ hybridization demonstrate intense positive expression of all three forms of NMDAR subunits, namely, NMDAR1 (Fig. 8a), NMDAR 2A (Fig. 8b), or NMDAR 2B (Fig. 8c) in the fusiform neurons of the MrD. It is of interest that the distribution of NMDAR 1 and NMDAR 2A mRNA is more widely distributed than that of NMDAR 2B mRNA in that the former are intensely present in the striatum and the globus pallidus in addition to the MrD whereas the latter is almost exclusively expressed in the fusiform neurons of the MrD and is absent from other parts of the neostriatum. In addition to the MrD, NMDAR1 was also found to be expressed in the granular cells of the Hip (Fig. 8d).

## Discussion

It is well known that the Hip plays an important role in learning and memory in general. However, increasing amount of evidence suggests that other brain regions such as the prefrontal cortex, amygdala, limbic system, and BNM are also involved in learn and memory [6–8]. Our previous studies have also identified a new subdivision of the neostriatum, MrD, to be involved in learning and memory, especially in associative learning and declarative memory [10, 19]. In this communication, we have compared the mechanism and the role of MrD and Hip in different forms of learning and memory and conclude that (1) the mechanism involved in non-declarative spatial learning and memory retrieval of non-declarative spatial memory for both the MrD and Hip is similar; (2) the roles of MrD and Hip in declarative long-term learning and memory function are deferent. MrD is likely to be involved in the entire process of long-term memory consolidation whereas the Hip only contributes to memory in the early stage; (3) NMDAR and pCREB are involved in memory function of both the Hip and the MrD with different mechanisms. The conclusion is based on the following observations: Firstly, in the Morris water maze test, the result of the escape latency which is a measurement of learning acquisition and the results of the crossing numbers during the spatial probe tasks, a measurement of memory retrieval for both the MrD-lesioned and the FF-transected rats, increases to the same extent suggesting that the mechanism involved in learning acquisition and memory retrieval for both the MrD and Hip is similar. Secondly, in the Y-maze test, when the lesions were made in rat brain immediately after 3-day training in the Y-maze, the performance of the MrD-injured and FF-transected rats was reduced to the same extent, suggesting that the MrD and FF were both involved in the early phase of the memory consolidation process. However, when the lesions were made 24 h after rats were trained in the Y-maze only, the MrD-injured rats but not the FF-transected rats performed more poorly than the sham-operated animals suggesting that the MrD may play a more important role than the Hip in the more advanced stage of the memory consolidation and retrial. This is consistent with the report of Zola-Morgan and Squire [20] that monkeys with hippocampal damage were severely impaired in their memory of recently learned objects, but they remembered objects learned long time ago similar to normal monkeys. Hence, they proposed that the Hip was required for memory storage for only a limited period of time after learning, so-called short-term memory [20] suggesting that the Hip is closely related to early phase of memory consolidation as demonstrated in this study. Electrophysiological studies have also shown that hippocampal LTP is attenuated by bilateral lesions in the MrD of neostriatum with kainic acid [21]. These observations suggested that the MrD may be involved in the entire process of memory consolidation that is from the initial stage to the more advanced stages of memory consolidation, such as long-term memory process involving memory storage and retrieval. Thirdly, NMDARs, pCREB, c-Fos, and c-Jun were found to be involved in memory consolidation of both the Hip and the MrD. However, NMDARs were found to regulate pCREB level in neurons of the MrD but not in neurons of the Hip suggesting that the mechanism underlying memory process in the MrD might be different from that of the Hip. Previous studies have shown that NMDARs are essential for the generation of LTP [22] and NMDA-receptor-dependent regulation of synaptic transmission in neurons represents an unifying mechanism for associative learning and declarative memory [13]. In addition, the CREB-dependent transcription in neurons has been demonstrated to be a crucial intracellular event for consolidation of long-term memory in several different organisms including Aplysia, Drosophila, mice, and rats [23]. CREB has also been regarded as a memory modulator and a molecular switch for memory formation. Protein kinase A-mediated phosphorylation of CREB and its product pCREB induce transcription of the immediate early genes which plays crucial roles in synaptic plasticity and is generally considered as an indication for neuronal activation [24–26]. Therefore, NMDARs, pCREB, and immediate early genes including c-Fos and c-Jun have been postulated to participate in the molecular process of learning and memory [27, 28].

In the present study, expressions of the NMDA receptor subunits 1, 2A and 2B, pCREB, and its target genes c-Fos and c-Jun were found to be markedly increased in the MrD of the neostriatum and the Hip after rats were trained in Y-maze for 3 days. Furthermore, the protein level of pCREB was increased in the fusiform neurons of the MrD and in the neurons of the Hip after treatment with OA which is an inhibitor of protein phosphatase 1 (PP-1), an enzyme responsible for dephosphorylation of pCREB. Moreover, when OA was directly injected into the MrD of rats, the performance of the rats in the foot-shock avoidance Y-maze test was markedly improved as compared with the control group. These results indicated that the pCREB plays an important role in learning and memory function of the MrD and the Hip. Interestingly, when the expression of pCREB in the MrD was suppressed by intraperitoneal injection of the NMDA receptor blocker, MK-801, the correct runs of the MK-801-treated rats in the Y-maze also decreased suggesting that both NMDARs and pCREB in the MrD of the neostriatum are involved in the learning and memory process and in retrial and that alteration of the expression of pCREB leads to changes of the learning and memory ability of rats as shown in the Y-maze test. The membrane receptor NMDARs, the nuclear transcription factor pCREB and the two immediate early genes c-Fos and c-Jun could potentially be involved in the same signaling pathway associated with long-term memory formation in the MrD of the rat neostriatum. NMDARs are however not involved in the regulation of pCREB expression in the neurons of the Hip since treatment with MK-801, a specific NMDAR blocker, did not affect the pCREB levels in the neurons of the Hip suggesting that the signaling pathway of learning and memory in the Hip may not be the same as the pathway in the MrD. Recently, we reported that the micro RNA (miRNA) expression patterns in the MrD is also distinct from that of the Hip, suggesting the role of miRNA in learning and memory function of the MrD probably is different from that of the Hip [29]. The results of miRNA also support the conclusion reported here that the signaling pathways of learning and memory functions between the MrD and the Hip could be different.

In summary, our results showed, firstly, that roles of both MrD and Hip involved in non-associative learning and non-declarative spatial memory are similar. Secondly, the MrD of the neostriatum is contributing to the entire process including both the early and late stages for the consolidation and retrieval of long-term memory formation whereas the Hip only related to the early stage of long-term memory formation and the short-term memory formation. Thirdly, NMDARs, pCREB, c-Fos, and c-Jun are involved in memory consolidation of both Hip and the MrD of the neostriatum. NMDARs might regulate pCREB level in neurons of the MrD of the neostriatum but not in neurons of the Hip. It may also suggest that the MrD of the neostriatum is involved in more complicated memory processes than the Hip.
